# Active microbial biofilms in deep poor porous continental subsurface rocks

**DOI:** 10.1038/s41598-018-19903-z

**Published:** 2018-01-24

**Authors:** Cristina Escudero, Mario Vera, Monike Oggerin, Ricardo Amils

**Affiliations:** 1 Centro de Biología Molecular Severo Ochoa (UAM-CSIC), Facultad de Ciencias, Universidad Autónoma de Madrid, 28049 Madrid, Spain; 2 Instituto de Ingeniería Biológica y Médica, Escuelas de Ingeniería, Medicina y Ciencias Biológicas, Departamento de Ingeniería Hidráulica y Ambiental, Escuela de Ingeniería, Pontificia Universidad Católica de Chile, Av Vicuña Mackenna, 4860 Santiago, Chile; 3 Centro de Astrobiología (CSIC-INTA), Ctra de Ajalvir km 4, Torrejón de Ardoz, 28850 Madrid, Spain

## Abstract

Deep continental subsurface is defined as oligotrophic environments where microorganisms present a very low metabolic rate. To date, due to the energetic cost of production and maintenance of biofilms, their existence has not been considered in poor porous subsurface rocks. We applied fluorescence *in situ* hybridization techniques and confocal laser scanning microscopy in samples from a continental deep drilling project to analyze the prokaryotic diversity and distribution and the possible existence of biofilms. Our results show the existence of natural microbial biofilms at all checked depths of the Iberian Pyrite Belt (IPB) subsurface and the co-occurrence of bacteria and archaea in this environment. This observation suggests that multi-species biofilms may be a common and widespread lifestyle in subsurface environments.

## Introduction

Microbial life is ubiquitous and diverse, even in subsurface environments^1^. Natural microbial communities most often live attached to surfaces or interfaces, forming biofilms, defined as the coexistence of one or more species of microorganisms sharing space in a self-produced matrix. The matrix is a three-dimensional structure mostly composed of extracellular polymeric substances (EPS) such as polysaccharides, proteins, nucleic acids, lipids and, above all, water. Therefore, the development of biofilms implies a change in genetic regulation and the consumption of energy to generate its components and maintain biofilm integrity^2–4^. However, biofilm lifestyle provides an ideal microenvironment where microorganisms can survive and grow even when external conditions are adverse. Some of the functions associated to biofilms are: adhesion to surfaces, retention of water, structuration of biomass, sorption of organic and inorganic compounds, enzymatic activity, nutrient source, redox regulation, or quorum sensing among others^5,6^.

Deep subsurface is considered an extreme environment characterized by darkness and anaerobiosis where the temperature and pressure increase with depth^1^. In these environments, geochemistry and geohydrology control nutrient and water availability and, therefore, the number and activity of microorganisms. As buried organic matter is scarce or no longer profitable, the principal source of substrates is virtually limited to mineral dissolution or abiotic processes that release energy from minerals. Thus, the main metabolisms operating in the deep subsurface are anaerobic and the energy obtained is low^7^. In addition, growth of microorganisms is influenced by rock porosity and the presence of fractures or faults in the system. Fractured rocks or those with high porosity present an increase in water flux and physical space which promotes microorganism colonization. Hence, most microorganisms should show very low metabolic rates or remain in a dormant state in the deep subsurface poor porous matrix substrates^8^. Consequently, it has been suggested that in these conditions microbial biofilms may not exist due to the high energetic cost required for their formation and maintenance^9^.

The ability of isolated subsurface microorganisms to form biofilms has been demonstrated *in vitro*^10^ as well as the formation of biofilms through *in situ* colonization experiments on added glass and rock surfaces in natural subsurface environments^11,12^. In addition, few studies have shown that microbial biofilms are formed in native rock matrixes at least in fracture zones, where the flux of water and nutrients is higher, or near the surface, where oxygen is present^13–15^. But up to now, there has been no information about the formation of native biofilms in deep poor porous rock matrix, where there is no oxygen, water is limited and life is supported mainly by anaerobic low energy metabolisms.

Fluorescence microscopy techniques are useful tools to study the three-dimensional structure of biofilms, but the reflection and autofluorescence of some minerals in rock samples make it very difficult to distinguish them from true positive signals^14^. Instead, other microscopy techniques such as scanning electron microscopy (SEM) have been applied, but no information about microbial or EPS composition of the biofilms was obtained^12,13,16^. Fluorescence *in situ* hybridization (FISH) techniques combined with fluorescence lectin-binding assay (FLBA) and other specific stains offer valuable information about biofilms^17^. FISH allows the identification of a particular living microorganism present in a sample due to the use of specific 16S rRNA probes and the study of their interactions using double hybridizations^18^. In addition, lectins labeled with fluorophores used in combination with other specific stains for DNA, proteins and lipids can provide data about the biofilm composition^17^.

In this work, samples from a devoted geomicrobiological drilling project (Iberian Pyrite Belt Subsurface Life detection, IPBSL), obtained in sterile and anaerobic conditions from cores at different depths, have been used to detect and characterize the microbial and chemical composition of native biofilms existing in poor porous rock samples from the deep subsurface of the Iberian Pyrite Belt by using FISH and FLBA coupled to confocal laser scanning microscopy (CLSM).

## Results and Discussion

### Fluorescence *in situ* hybridization on mineral substrates

The principal problem when applying fluorescence techniques in rock samples is the presence of minerals. On the one hand, their intrinsic fluorescence can hinder the detection of true signals and, on the other hand, as in any other substrate, probes and dyes can bind unspecifically. Thus, the correct choice of fluorophores and dyes is essential for each rock sample.

Since not all minerals reflect light or present fluorescence at the same wavelength, each rock sample was checked by fluorescence microscope to select suitable fluorophores to be used in further experiments, avoiding mineral fluorescence interference. To make sure that the observed fluorescence signal in hybridization experiment was actually biological, additional criteria were taken into account: the use of DNA-binding dyes 4′,6-diadimino-2-phenylindole (DAPI) or Syto-9 as counterstaining as well as the form, size and emission spectrum of the signal.

An additional potential problem when applying FISH techniques to rock samples is the unspecific binding of the probe or dye to the inorganic surface resulting in high background or false positive signals. Additional controls were carried out using a NON338 probe with different fluorophores and with each dye in clean, sterilized rock samples in which no organic matter was detected by Raman spectroscopy (data not show). In this case, any detected signal is due to the unspecific binding of the probe to the mineral substrate. Each selected dye and fluorophore showed some degree of nonspecific binding in some of the samples. This heterogeneous unspecificity of the probes may be due to the different charge or hydrophobicity that each one presents (Table 1), resulting in diverse Van der Waals or hydrophobic interactions with the array of minerals in the native samples (see Supplementary Table S1)^19^. All dyes or fluorophores used on each sample were chosen to avoid both non-specific binding with the mineral substrate and mineral fluorescence interference.

**Table 1:** Summary of dyes and fluorophores used in this study. Net charge and LogD were calculated using the molecular structure of the fluorophore with hydrolyzed reactive group. The molecular structure of Syto9 and Sypro ruby is not available.

Fluorophore	Excitation wavelength	Emission wavelength	Technique	Net charge pH 7,4	LogD at pH 7,4
Pacific Blue	410	455	CARD-FISH/DOPE-FISH	−1,96	−3,37
FITC	490	525	FLBA	−1,99	−1,48
Alexa 488	490	525	CARD-FISH	−2,99	−10,13
Alexa 594	590	617	CARD-FISH	−2,99	−7,04
CY3	550	570	FISH/DOPE-FISH	0,00	4,69
DAPI	350	461	DNA stain	2,00	−4,68
SYTO9	485	500	DNA stain	—	—
Nile red	552	636	Lipids stain	0,00	3,83
SYPRO ruby	280/450	610	Proteins stain	—	—

### Improving microbial biofilms detection

**Figure 1:**
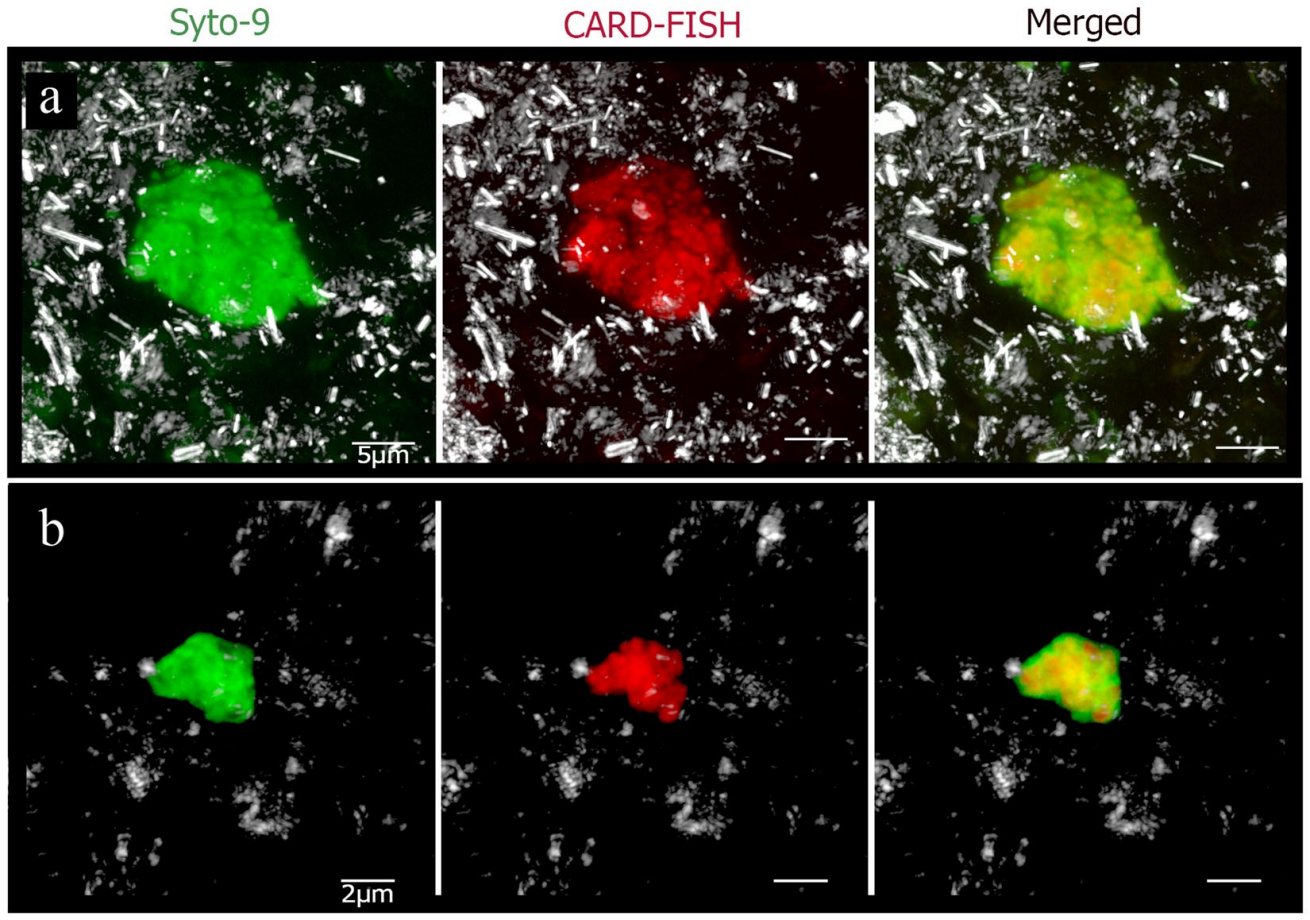
Detection of bacteria and archaea in subsurface drilled samples from different depths. SYTO-9 stain (green), reflection (gray) and CARD-FISH (red) of (**a**) bacteria at 420 mbs and (**b**) archaea at 496,8 mbs. Scale bars, 5 μm (**a**) and 2 μm (**b**).

With the aim of detecting and identifying the maximum amount of microorganisms and studying their distribution, CAtalyzed Reporter Deposition-FISH (CARD-FISH) was chosen. The amplification of the probe signal by CARD-FISH allows the detection of microorganisms even when the number of their ribosomes is rather low^20^, which is to be expected in deep subsurface environments due to limited energy supply and low microbial metabolic rates^21^. Using domain-level probes, a high number of living Bacteria and Archaea were detected along the column, usually forming compact colonies (Fig. 1).

**Figure 2:**
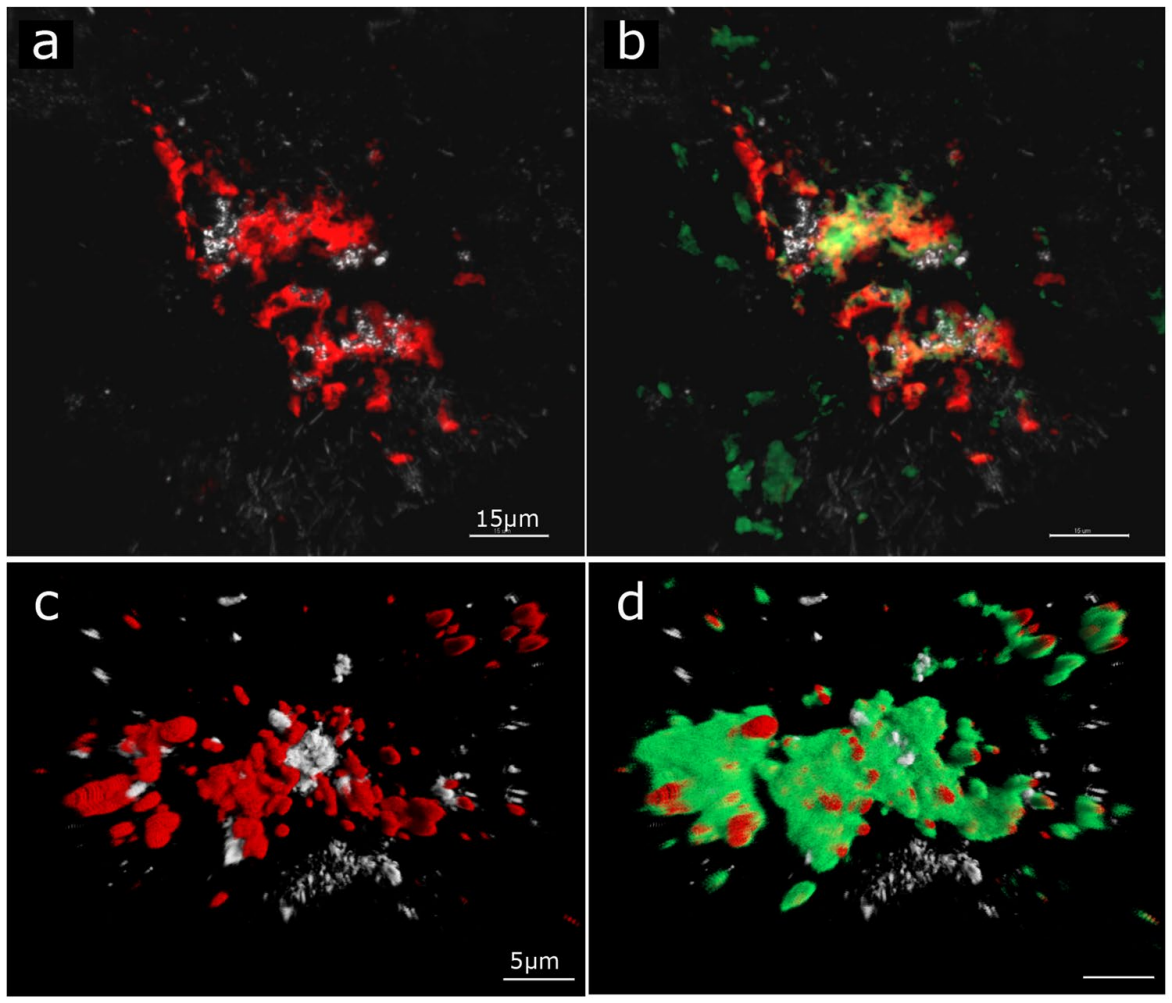
Bacterial biofilms detected at 414,8 mbs by CARD-FISH (**a** and **b**) and FISH (**c** and **d**). In red, EUB338 I-III probe signal; in green, FITC-ConA lectin signal. Scale bar: a and b 15 μm, c and d 5 μm.

CARD-FISH combined with FLBA was applied to deep subsurface samples of the IPB to determine the presence of natural microbial biofilms in the rock matrix. Different lectins were tested (Table 2) and parallel hybridizations were carried out in clean rock controls with no signal detection. CARD-FISH and FLBA analysis revealed the presence of living bacterial and archaeal microcolonies surrounded by traces of polysaccharides at all checked depths (Fig. 2a,b). It was detected the presence of α-linked fucose residues and galactosyl (β-1,3) *N*-acetylgalactosamine residues in some of the biofilms visualized through AAL, UEA I or PNA lectins respectively. Nevertheless, Concanavalin A (Con A), which specifically binds internal and non reducing α-D glucosyl and α-D mannosyl residues, was the lectin that revealed broader biofilm surface. However, the lectin signal was poor and scarce, even when more than one lectin was used to reveal the biofilm structure. This fact may suggest that either i) the existence of certain glycoconjugates unrecognized by the lectin used; ii) that in the subsurface, EPS production may be reduced in response to low nutrient levels^22^; or iii) the signals correspond to the remains of the exopolysaccharides which were consumed by microorganisms, since the EPS matrix can serve as a reservoir of nutrients to maintain the geobiochemical cycles^23,24^. Furthermore, because CARD-FISH requires a large and aggressive sample preparation protocol, the low and sparse lectin signals observed could be also due to the numerous washing steps, the inactivation of peroxidases or the cell permeabilization steps required by this technique.

**Table 2:** Summary of lectins and their specificity used in this study.

Name	Abbreviation	Source	Specificity
Concanavalin A	ConA	Jack beam (*Canavalla ensiformis*)	Glucose, Mannose
Aleuria aurantia lectin	AAL	*Aleuria aurantia*	Fucose
Peanut agglutinin	PNA	Peanut (*Arachis hypogea*)	Galactose
Ulex europaeus I	UEA I	Furze gorse (*Ulex europaeus*)	Fucose

To avoid the influence of the CARD-FISH protocol on the integrity of the biofilms, we repeated the experiment using FISH for microorganism detection. The FISH-FLBA hybridization showed the existence of well conserved and mature biofilms on the subsurface rock matrix (Fig. 2c,d). However, the number of colonies visualized by FISH was lower, as expected, than by CARD-FISH. This reduction in the number of microorganisms detected in comparison to CARD-FISH may be due to the low metabolic rate that some microorganisms present in the subsurface, since FISH signal intensity is directly proportional to the number of ribosomes present in the cells^25^. Furthermore, the microorganisms that comprise these biofilms detected by FISH are not in a dormant state but are metabolically active.

**Figure 3:**
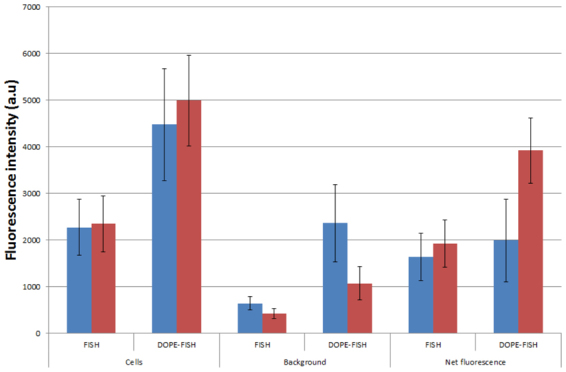
Comparison of FISH and DOPE-FISH mean fluorescence intensity of cells, background and net fluorescence efficiencies in *E. coli*. Hybridizations were carried out with FISH buffer (blue) or geneFISH buffer with pre-hybridization step (red).

In order to visualize as many microorganisms as possible without compromising the integrity of biofilms, Double Labeling of Oligonucleotide Probes (DOPE)-FISH^26^ was checked as an alternative signal amplification method. The signal intensity of DOPE-FISH was compared with FISH signal using *E. coli* in laboratory control experiments (Fig. 3). These results showed that the fluorescence signal using DOPE-FISH was almost twice that of FISH, in accordance with Stoecker, *et al*.^26^. However, DOPE-FISH background was 3.7 times higher than that of FISH, resulting in a final increase of just 1.2 times in net fluorescence signal compared to FISH, defined here as cell fluorescence minus background fluorescence, when the hybridization was carried out in the same conditions. To increase the signal-noise ratio, alternative hybridization buffers were tested. It has been described that the CARD-FISH buffer, which contains blocking reagent and dextran sulfate, increase the signal up to 20%^27^. GeneFISH hybridization buffer^28^, which contains extra blocking reagents such as salmon sperm DNA and yeast RNA to decrease the background, was also tested. Our results indicate that the use of geneFISH buffer in a pre-hybridization incubation as well as in the hybridization not only decreased the background intensity but increased the cell signal intensity, yielding an increase of net fluorescence signal in DOPE-FISH of 2.4 times over that of FISH (Fig. 3). Other methods for signal amplification such as MIL-FISH (Schimak *et al*.^27^) were tested but no remarkable improvement was achieved in our samples.

### Biofilms in deep subsurface rock matrix

**Figure 4:**
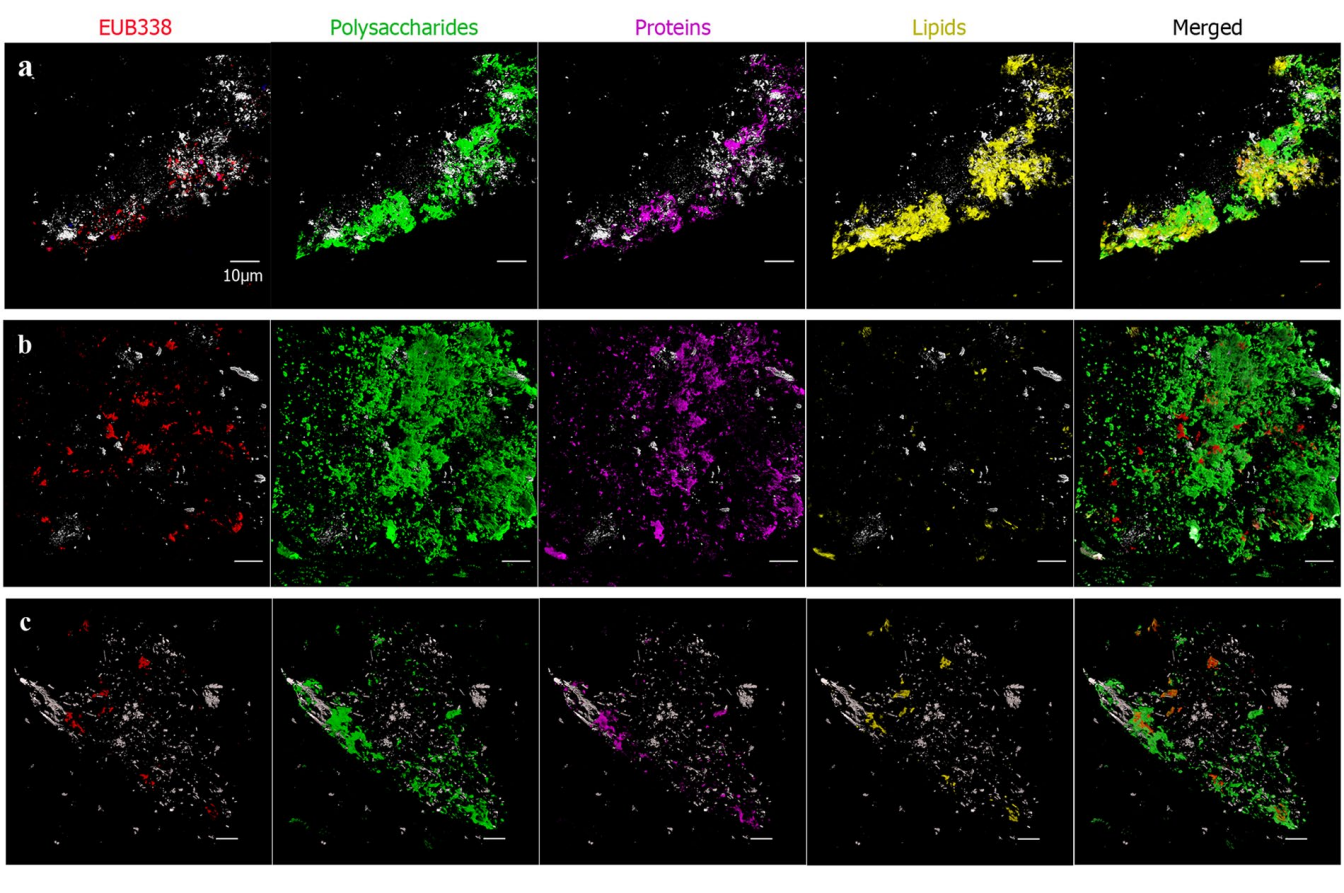
Bacterial biofilm detection in subsurface hard rock samples from different depths. DOPE-FISH of bacteria (red), FITC-ConA (green), SYPRO ruby (violet) and Nile red (yellow) at 355,7 mbs (**a**), 420 mbs (**b**) and 519, 1 mbs (**c**). Scale bar 10 μm.

DOPE-FISH and FLBA were then applied to subsurface rock samples showing a greater number of detected microorganisms than FISH hybridizations with a similar degree of biofilm integrity (Fig. 4). Proteins and lipids are also present in the subsurface biofilms. In most of the detected biofilms, the main detected components were polysaccharides and proteins (Fig. 4b,c), with some exceptions where lipids seemed to be more abundant than proteins (Fig. 4a). All colonies exhibited, at least, traces of EPS surrounding them. In fact, it is noticeable that biofilms were detected in samples from all checked depths, even in poor porous substrates. This indicates that the biofilm lifestyle is common in the subsurface despite being considered an oligotrophic environment along with the energetic cost of biofilm production and maintenance^3,4^. In an environment where water and nutrients are limited and energy must be obtained from inorganic sources, the derivation of energy to biofilm production underlines its importance not only in the retention of nutrients and water^29^ but also in efficiency in the generation of energy^30^.

### Multi-species biofilms in deep subsurface rock matrix

**Figure 5:**
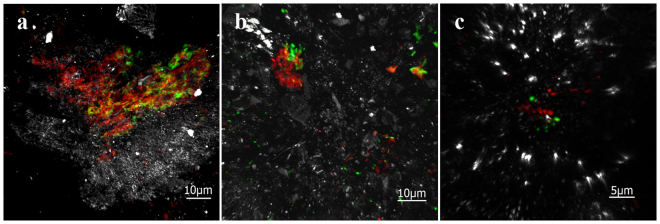
Double CARD-FISH detection of bacterial and archaeal mixed colonies in drilled samples from different depts. Double CARD-FISH with bacteria probe (green) and archaea probe (red) at (**a**) 139, 4 mbs, (**b**) 284 mbs and (**c**) 414, 8 mbs. Scale bars, a and b 10 μm, c 5 μm.

**Figure 6:**
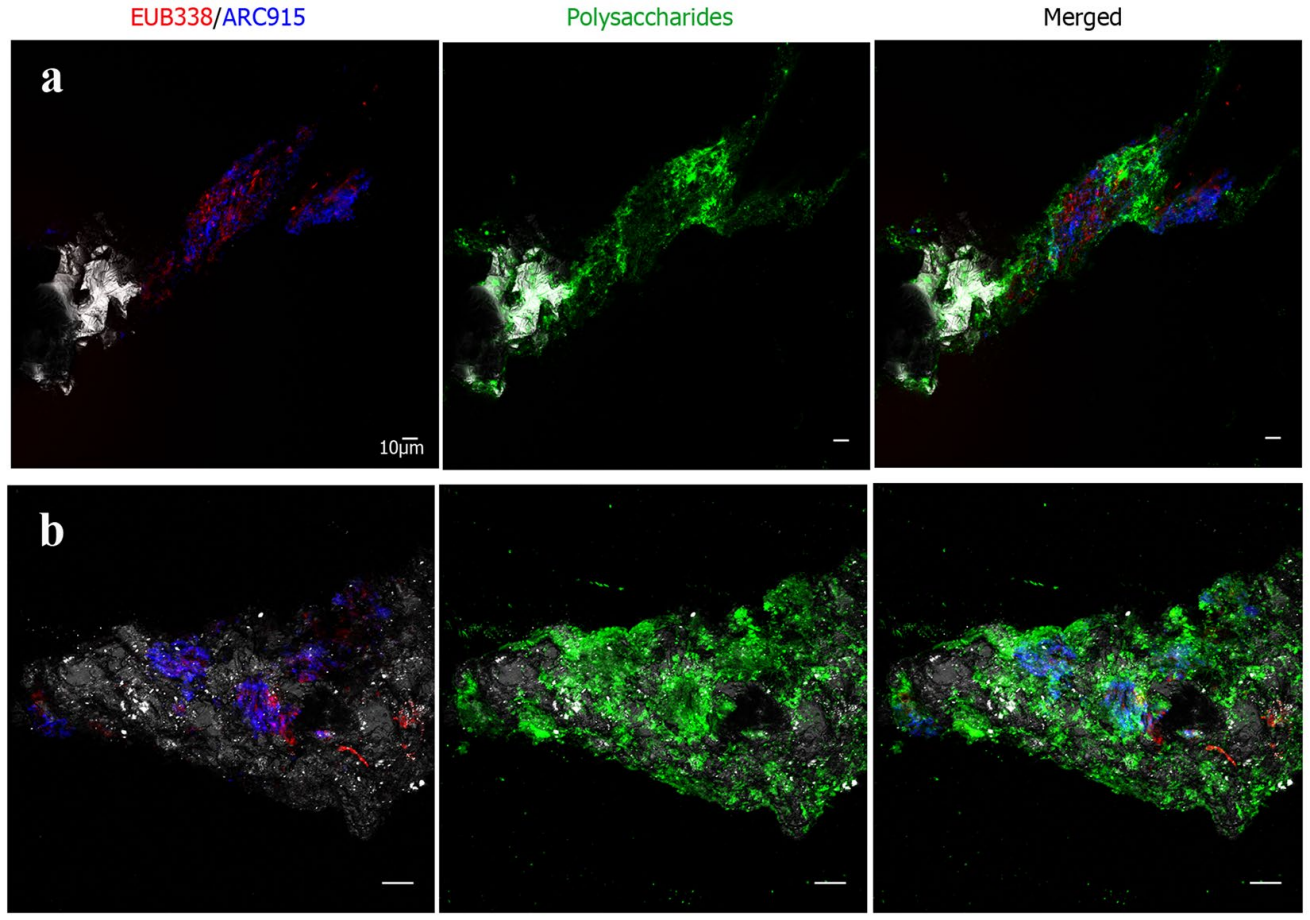
Detection of bacterial and archaeal mixed biofilms using double DOPE-FISH and FLBA. DOPE-FISH with bacteria (red) and archaea (blue) probes and FLBA with ConA, AAL and PNA lectins (green) at 139,4 mbs (**a**,**b**). Scale bar 10 μm.

In some samples, DNA stain signals were more abundant than the correspondent bacterial or archaeal FISH signals (Fig. 1), which can be related to the existence of mixed colonies of both types of microorganisms. To corroborate whether mixed colonies are present in the IPB subsurface, we first tried double CARD-FISH using bacterial and archaeal probes to visualize all living prokaryotes of the system (Fig. 5). Because bacterial and archaeal mixed colonies were detected in some of the samples from different depths, double DOPE-FISH and FLBA were used to determine whether these microorganisms were able to produce biofilms (Fig. 6). Figure 6 shows the existence of native subsurface biofilms with a mixture of microorganisms from both domains. Previous studies had described syntrophic consortiums of bacteria and archaea in anoxic sediments promoting the anaerobic oxidation of methane^31,32^. Other studies have shown the co-occurrence of microorganisms from both domains in a broad range of habitats which are important for the maintenance of biogeochemical cycles such as the iron, sulfur, nitrogen or carbon cycles^33–37^. In most cases, the structural relationship between both kinds of microorganisms is still unknown. However, the existence of these multidomain biofilms is indicative of the advantage of bacterial and archaeal collaboration^38^ which may be extremely critical on the subsurface. Futures studies should be conducted to identify these microorganisms and the nature of their association in the subsurface of the IPB.

It is interesting to note that usually the EPS signal is not concentrated in only one single colony but extends along the substrate matrix, interconnecting more than one cluster of cells (Figs 4 and 6), separated by a substantial distance. Gantner, *et al*.^39^ showed that the “calling distance” of quorum sensing can extend up to 78 μm between single species biofilms. However, cooperation between different microorganisms seems to need their co-aggregation^38,40,41^. Yet, in subsurface environments, where confined space can limit the aggregation of cells, the possibility of communication and cooperation by diffusion of metabolites between different microcolonies, even when the distance is significant, should not be discarded.

Several questions still remain: how general is this structural strategy in the deep subsurface or how important is it to the efficient operation of the biogeochemical cycles in these restrictive environments. We should keep in mind the advantage offered by specialized microniches with different optimal conditions in a solid matrix, like the hard rock subsurface, which need to be interconnected to interchange metabolic products, thus generating a network of specialized metabolisms which would be impossible in a liquid world and difficult in a soft sedimentary system. To answer these questions we need to identify the microorganisms participating in these biofilms. The use of specific fluorescent probes should help to solve these queries. The main limitation of FISH and FLBA is the choice of the appropriate probes or lectins, which can be solved by previous genomic or biochemical analysis. Conversely, these techniques may offer faster global data about the ecosystem but provide no information about its distribution in the solid substrate matrix. Microscopy techniques, in spite of being time consuming, make it possible to analyze the subsurface ecosystem at the microniche level, allowing the study of microbial and EPS composition and distribution of existing biofilms. Because all techniques have limitations, the combination of several techniques to study deep subsurface life will be essential. Within these techniques, FISH and FLBA are powerful tools to be considered.

## Methods

### Sampling and sample processing

Sampling, mineralogical (XRD) and elemental analysis (TXRF) were carried out as described^42^. Samples were fixed with 4% formaldehyde for 2 h at 4 °C and stored in phosphate-buffered saline (137 mM NaCl, 2.7 mM KCl, 10 mM Na_2_HPO_4_ and 1.8 mM KH_2_PO_4_, pH 8): ethanol (1:1) at −20 °C until further processing. As controls, subsurface rocks of the same depths of the samples studied were used. Controls were made by cleaning and sterilization as described^43^. Under sterile conditions, samples were crushed with a mortar to the size of grains of sand, embedded in 0.2% agarose (Conda, Spain) and stored at −20 °C until further processing.

### Log D calculations

Log D and log P values of each fluorophore and dye were calculated in MarvinSketch 16.9.12 (Chem Axon, Cambridge, MA) using the structure of hydrolyzed reactive group as described^44^.

### Fluorescence *in situ* hybridization

CARD-FISH experiments were performed as previously described in detail by Pernthaler, *et al*.^20^, with minor modifications. For cell wall permeabilization, samples were treated with lysozyme and achromopeptidase solutions. Endogenous peroxidases were inactivated as described^45^. Hybridization was performed with 5′-HRP-labeled oligonucleotide probes (Biomers, Ulm, Germany) for 2 h at 46 °C and then samples were washed at 48 °C for 10 min. Stringencies were regulated for each probe by adjusting formamide (FA) and NaCl concentration in hybridization and washing buffer respectively: EUB338 I-III mix probes^46,47^, 35% FA (vol/vol), 0,08 M NaCl; ARC915^48^, 20% FA (vol/vol), 0,225 M NaCl; NON338^49^, 0% FA (vol/vol), 0,9 M NaCl. Tyramide signal amplification was carried out for 45 min at 46 °C. In double CARD-FISH experiments, an additional inactivation of peroxidases was done between hybridizations.

FISH was performed in subsurface rock samples as described by Glöckner, *et al*.^50^ using Cy3 single-labeled EUB338 I-III mix probes (Biomers, Ulm, Germany).

Single- and double- Cy3 labeled EUB338-I probes (Biomers, Ulm, Germany) were compared using *E. coli* pure-culture. *E. coli* DH5α was grown in Luria-Bertani medium (10 g/l trytone, 5 g/l yeast extract and 5 g/l NaCl). Cells were harvested during logarithmic growth phase, fixed in 4% formaldehyde for 2 h at 4 °C and concentrated using 0.2 μm polycarbonate membrane filters (Millipore, Germany). FISH and DOPE-FISH were carried out with identical hybridizations and washing buffers^50^, as well as identical hybridization (2 h) and washing (10 min) times in order to compare the effect of adding fluorophores to the probe. To decrease background intensity in DOPE-FISH experiments, FISH and geneFISH hybridization buffers were compared. GeneFISH buffer was prepared as described^28^. An additional incubation with geneFISH buffer was carried out without probe for 1 h at 46 °C previous to the hybridization. All experiments were carried out in triplicate.

DOPE-FISH was performed in subsurface rock samples permeabilized with lysozyme as described by Pernthaler, *et al*.^20^, using geneFISH buffer for pre-hybridization and hybridization step.

### EPS staining

Polysaccharides were visualized by Fluorescence Lectin Binding Assay (FLBA), using lectins conjugated with fluorescein isothiocyanate (FITC) fluorophore (Vector Laboratories, Burlingame, CA, USA, Table 2). Lectins were diluted using the appropriate buffer suggested by the manufacturer. Samples were washed with lectin specific buffer and stained as described by Zhang, *et al*.^51^. Lectins were employed alone or in combination as described^52^.

Proteins were stained with SYPRO ruby (Thermo Fisher, USA) prior to FLBA. Samples were incubated with the stain for 30 min and washed three times with filter-sterilized milliQ water. Lipids were stained adding 1 µg/ml Nile red (Merck, Germany) in a mix of 1:4 Vectashield (Vector Laboratories, Burlingame, CA, USA): Citifluor (Citifluor, London, United Kingdom).

### Counterstaining and mounting

Samples were counterstained with DAPI (4′,6-diadimino-2-phenylindole) or Syto9 (Thermo Fisher Scientific, USA) according to manufacturer instructions and covered with the Vectashield: Citifluor mixture. Subsurface samples were mounted in µ-slides 8 wells glass bottoms (Ibidi, Germany).

### Microscopy

Samples were imaged in the Optical and Confocal Microscopy Service of the Centro de Biología Molecular Severo Ochoa (Madrid, Spain) using a confocal laser scanning microscope LSM710 coupled with an inverted microscope AxioObserver (Carl Zeiss, Jena, Germany) and equipped with diode (405 nm), argon (458/488/514 nm) and helium and neon (543 and 633 nm) lasers. Images were collected with a 63×/1.4 oil immersion lens.

Lambda-mode was used to individually characterize the emission spectral signature of every fluorophore and dye used in the experiments and to determine the source of the signal fluorescence in the rocks hybridizations. Only the signals that matched the specific emission spectrum of each used fluorophore were accepted as positive signals.

To compare the fluorescence signal intensities in FISH and DOPE-FISH experiments, images were taken with the same confocal microscope settings. At least 3000 individual cells were analyzed in each experiment. The mean fluorescence of microorganisms and background in *E. coli* controls were quantified with Fiji software^53^. The net fluorescence in *E. coli* controls was considered to be the result of the mean fluorescence of the microorganisms less the mean fluorescence of the background.

Biofilm images were further processed using Imaris 7.4.software (Bitplane AG, Zurich, Switzerland).

## Electronic supplementary material

Supplementary Dataset 1
